# Supplementary material for: Elucidation of Focal Adhesion Kinase as a Modulator of Migration and Invasion and as a Potential Therapeutic Target in Chronic Lymphocytic Leukemia
Source: Cancers (Basel). 2022 Mar 22;14(7):1600. doi: 10.3390/cancers14071600 (PMC8996841; doi:10.3390/cancers14071600)
Supplement: Supplementary file 1 [file cancers-14-01600-s001.zip › cancers-1600441-supplementary.pdf]

cancers MDPI

Article

Elucidation of Focal Adhesion Kinase as a Modulator of Migration and Invasion and as a Potential Therapeutic Target in Chronic Lymphocytic Leukemia

Thomas A Burley [1], Andrew Hesketh [2], Giselda Bucca [2], Emma Kennedy [1], Eleni E. Ladikou [1,3], Benjamin P. Towler [1], Simon Mitchell [1], Colin P. Smith [2,4], Christopher Fegan [5], Rosalynd Johnston [3], Andrea Pepper [1,*,†] and Chris Pepper [1,†]

Supplementary Figures

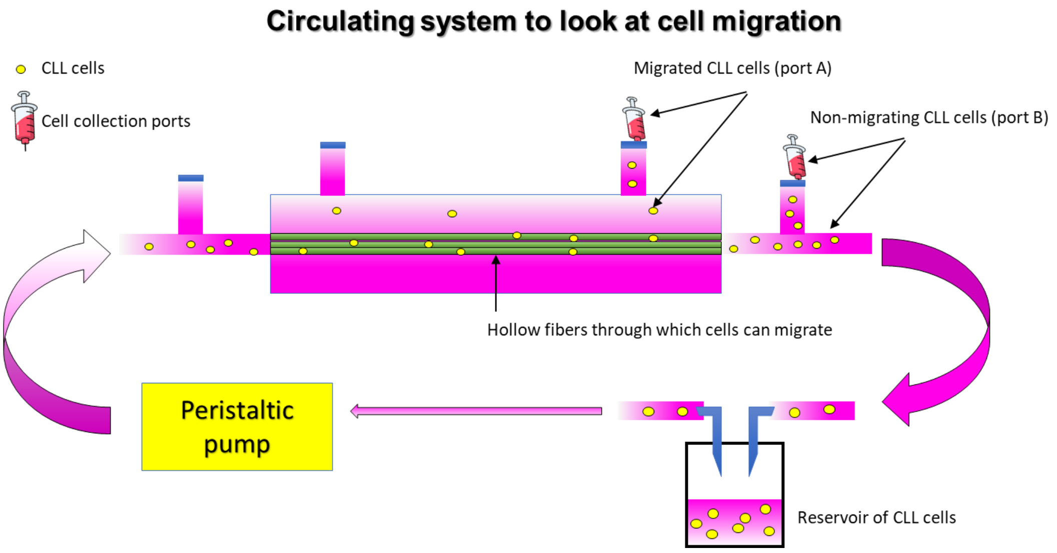

Figure S1. Schematic of the circulatory system.

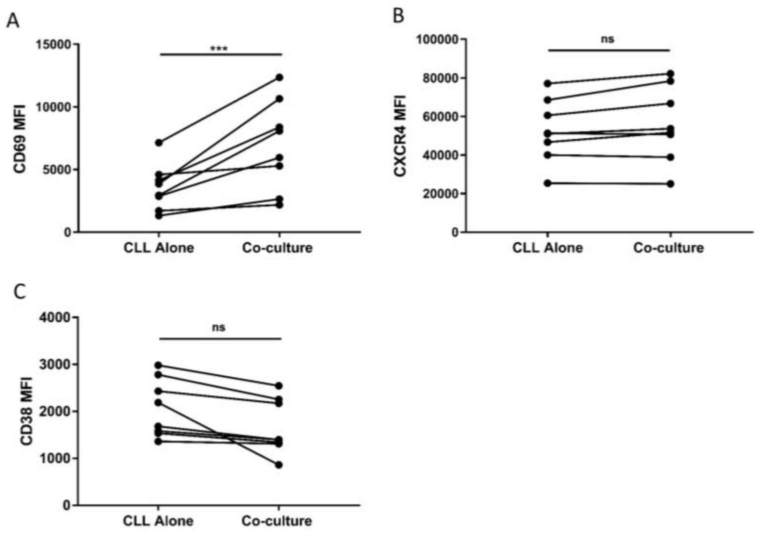

Figure S2. CD40L Fibroblast co-culture impact on CLL phenotypic markers after 4h. CLL phenotypic marker level MFIs, with or without CD40L fibroblast co-culture were quantified by flow cytometry on CD5+ CD19+ gated cells. CLL samples from the same patient are connected by a line. *** $p \leq 0.001$.

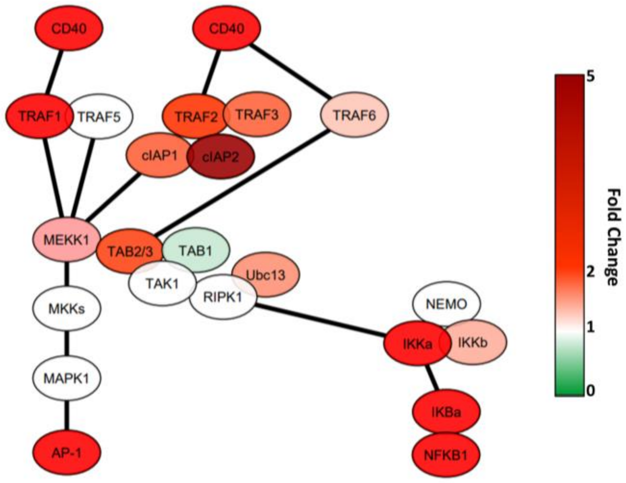

Figure S3. CD40L fibroblast co-culture upregulated CD40 signaling pathway proteins in CLL cells. RNA-seq differential expression overlayed on a CD40 signaling pathway cytoscape network.

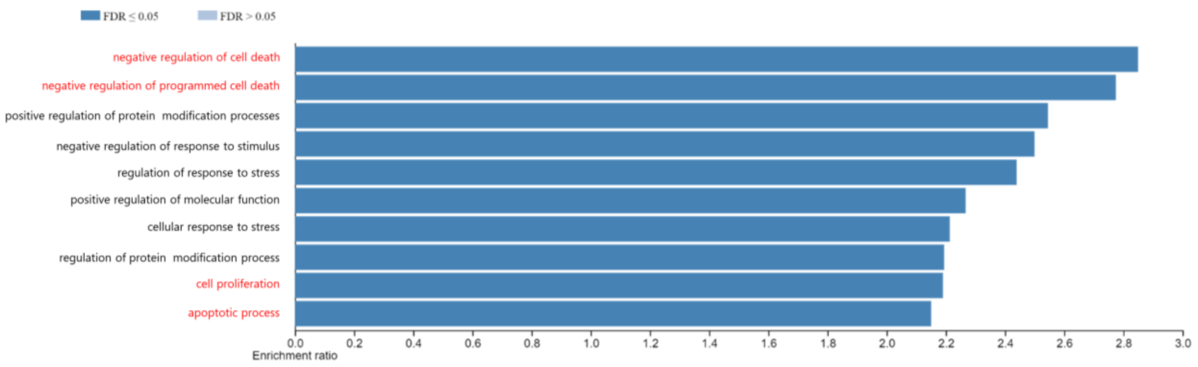

Figure S4. Common upregulated genes between the *in vitro* and co-culture system. The top 10 overrepresented pathways in the overlap between differentially expressed genes in the *vitro* system migratory cells and the co-culture system in the KEGG pathway database.

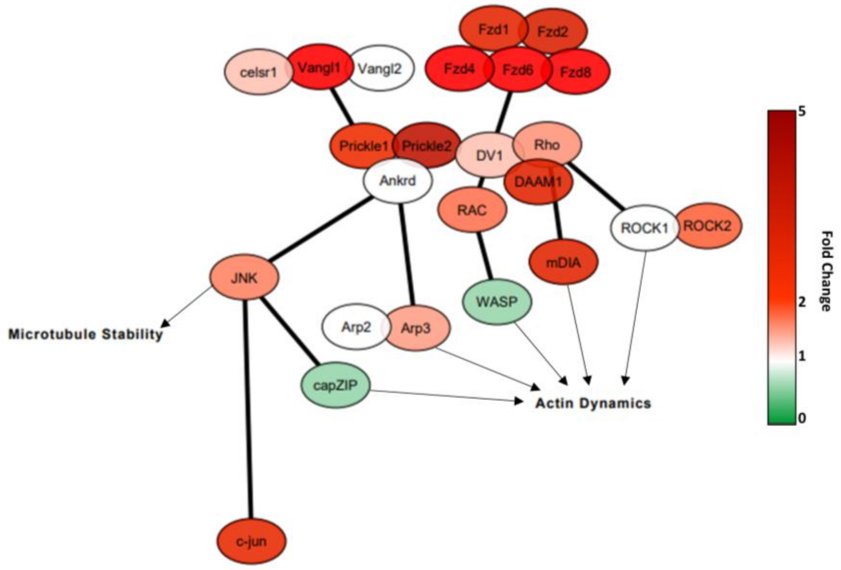

Figure S5. CLL migratory cells upregulated the WNT/PCP pathway. RNA-seq differential expression of migratory CLL cells overlayed on a WNT/PCP signaling pathway cytoscape network.

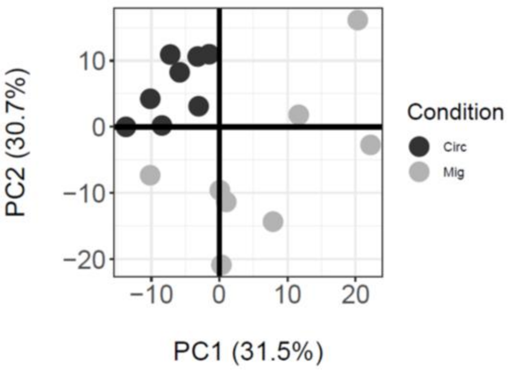

Figure S6. Principal component analysis (PCA) of CLL patient miRNA-seq data comparing circulatory and migratory CLL patient samples isolated from the *in vitro* system.

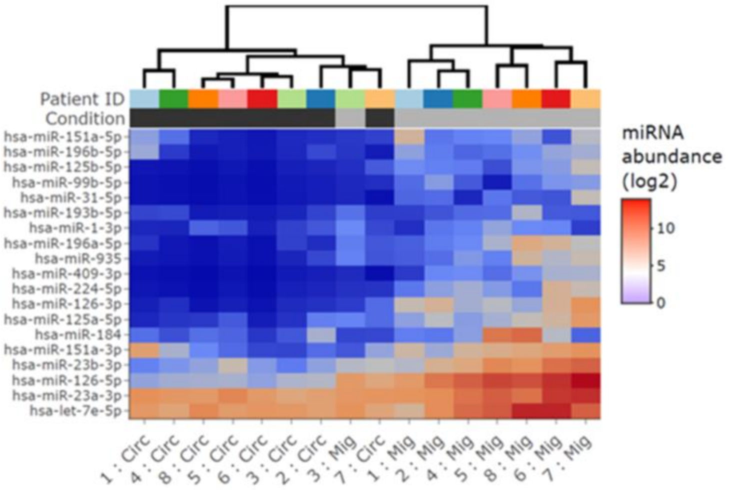

Figure S7. Hierarchical clustering of the circulatory vs migratory paired miRNA-seq samples for the 8 CLL in vitro system patients on the 19 differentially expressed miRNAs.

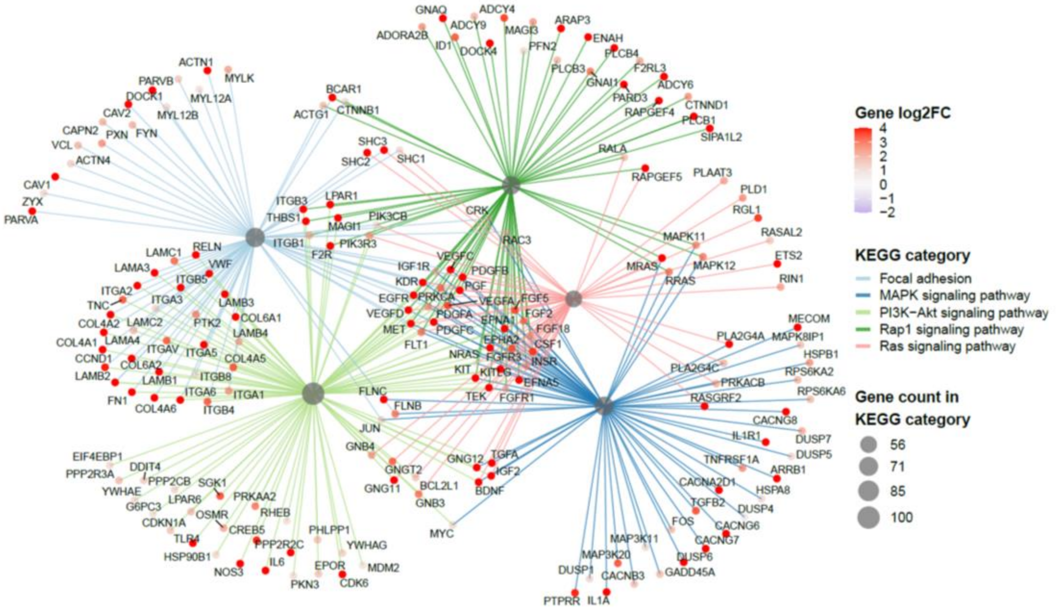

Figure S8. Pathway level analysis illustrating the up-regulated transcriptome KEGG pathways and upregulated GOA mRNA targets of the miRNAs.

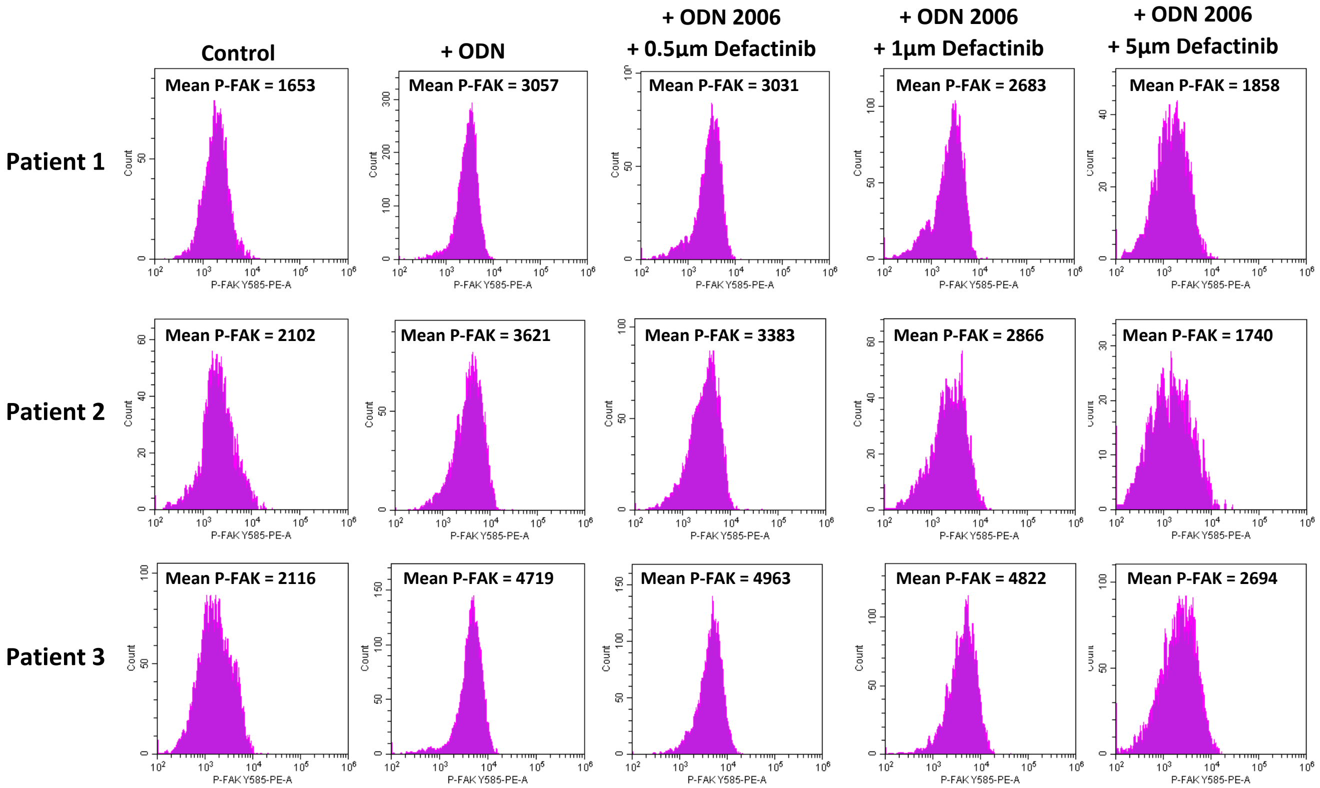

Figure S9. Stimulating CLL cells through TLR9 causes an increase in p-FAK. PBMCs from 3 different CLL patients were incubated with or without ODN2006 and for 24h in triplicate and the p-FAK levels assessed by flow cytometry. The mean fluorescence intensity was determined for both groups.

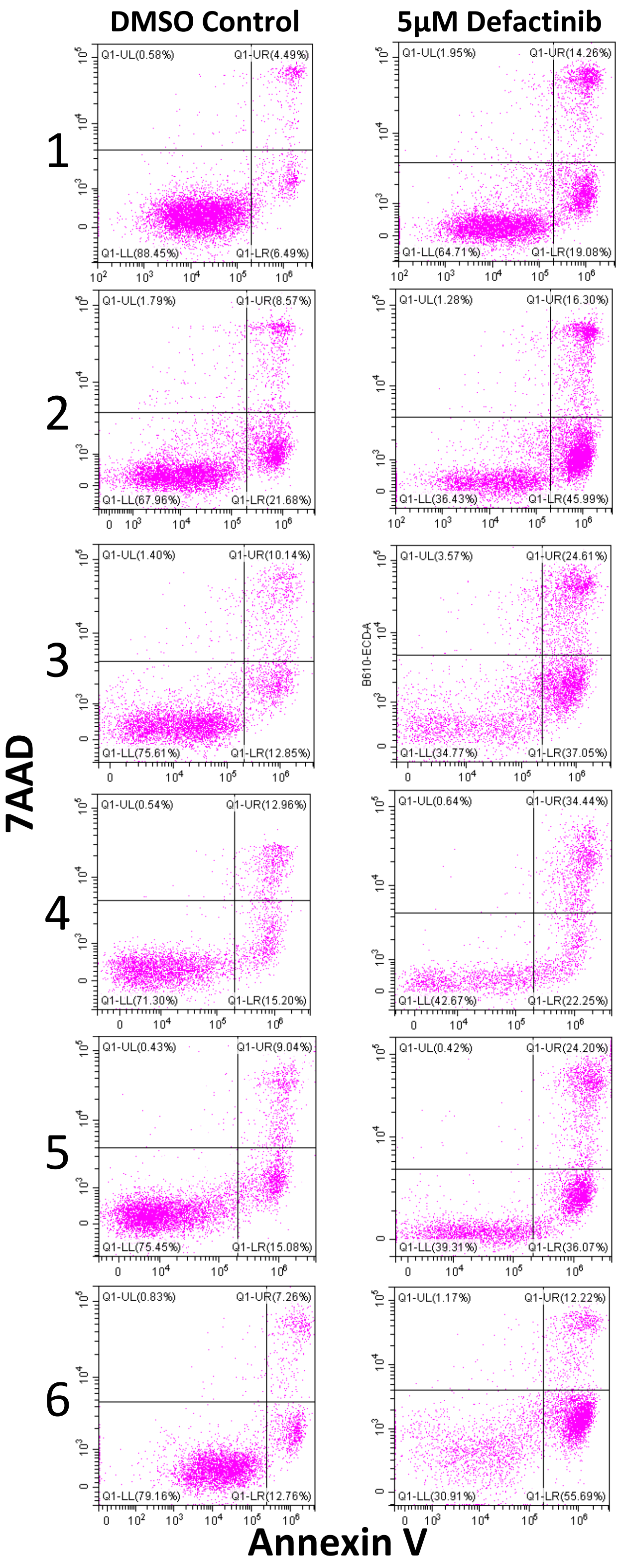

Figure S10. Defactinib treatment induces some cell death. PBMCs from 6 different CLL patients were incubated with ODN2006 and 5µM Defactinib for 24h and the levels of apotptosis was assessed by 7AAD/Annexin V staining.

% Migration Inhibition (vs DMSO Ctrl)	% Normalized Apoptosis (vs DMSO Ctrl)
80.7	44.2

Figure S11. Migration vs Cell viability. Mean % migration from the transwell experiments against cell viability for PBMCs from 6 different CLL patients after incubation with ODN2006 and 5μM Defactinib for 24h.

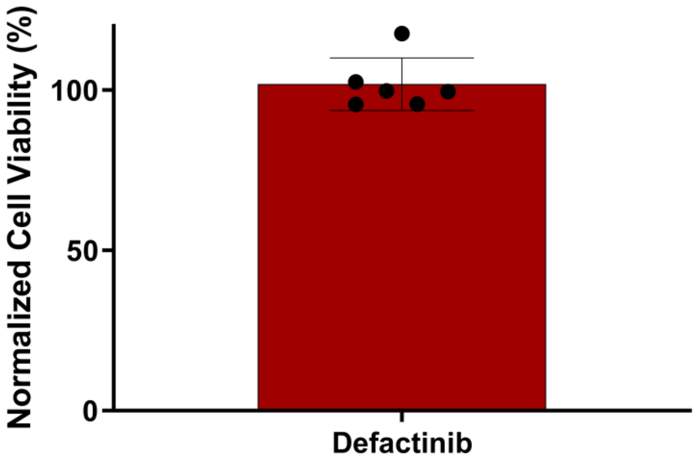

Figure S12. Invasion assay cell viability. PBMCs from 6 patients were pre-treated for 2h and 7AAD/Annexin V staining was performed to assess cell viability at the time of invasion assay initiation. Normalized to control cell viability.

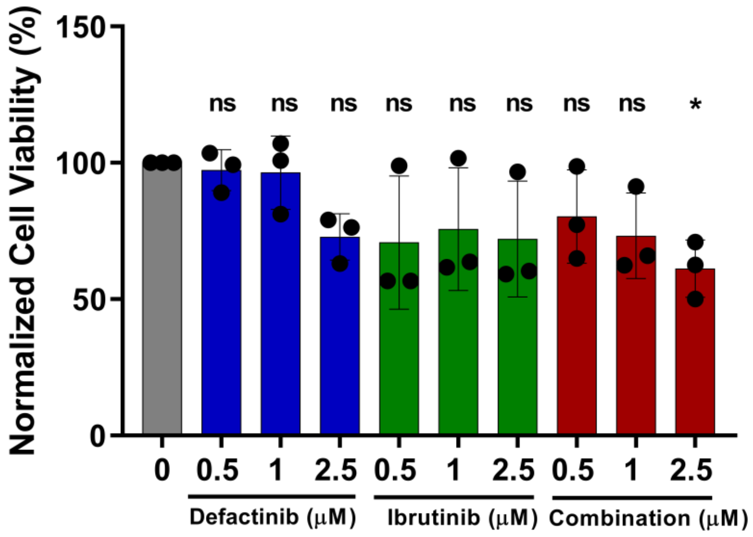

Figure S13. Cell viability following defactinib and ibrutinib treatment. PBMCs from 3 different CLL patients were incubated with defactinib, ibrutinib or a combination of both (molar ratio 1:1) for 24h before assessing the cell viability by 7AAD/ Annexin V staining.

Supplementary Methods

Sample Processing

Peripheral blood mononuclear cells (PBMCs) were isolated by Histopaque®-1077 (Sigma-Aldrich) density gradient centrifugation.

In Vitro Circulatory System

The insides of the Polysulfone hollow fibres were coated with gelatin to allow the adhesion of endothelial cells (10 X 10^6 cells added) and allowed to attach for 3 hours at 37°C before initiating circulation of 40 mL M199 medium supplemented with 20% fetal bovine serum at low level of shear force overnight (1.5 dynes/cm^2). The next day the shear force was increased in increments until a final shear force of 15.5 dynes/cm^2 was achieved. CLL cells were added to the reservoir in medium supplemented with recombinant human interleukin 4 (5 ng/mL) (R&D Systems) and circulated through the system for 24 h.

RNA isolation and quantitative real-time PCR (qRT-PCR)

Total RNA was isolated using Qiazol and RNeasy kit (Qiagen) according to the manufacturer's instructions. RNA was reverse transcribed to cDNA using the High-Capacity cDNA Reverse Transcription Kit according to manufacturer's instructions (Applied Biosystems) and mixed with the TaqMan Fast Advanced Master Mix (Applied Biosystems). For the qPCR the ptk2 (Hs01056457_m1) and 18S rRNA Endogenous Control Taqman gene expression assays (Applied Biosystems) were used. qPCR was performed in triplicate in three independent experiments using the AriaMx Real-time PCR System (Agilent) and the thermal profile: 50°C for 2 min, 95°C for 2 min, and then 40 cycles of 95°C for 1 s and 60°C for 20s. Expression data was normalized to the geometric mean of the house keeping gene and the fold change calculated using the Agilent Aria MX software.

Supplementary Tables

Table S1. Antibodies used.

Antibody	Conjugate and Clone	Supplier
Anti-CD69	Brilliant Violet 785 / FN50	Biolegend
Anti-CD62L	Brilliant Violet 650 / DREG-56	Biolegend
Anti-CXCR4	PerCP/Cyanine5.5 / 12G5	Biolegend
Anti-CD38	APC / HB-7	Biolegend
Anti-p-FAK	PE / K73-480	BD Biosciences

Table S2. A–F. Immunophenotypic characterization of the patient samples used in each experiment.

A

RNA-Seq Circ vs Mig							
**Sample ID**	**CXCR4 %**	**CXCR4 MFI**	**CD49d %**	**CD49d MFI**	**CD38 %**	**CD38 MFI**	**IGHV Status**
Patient 1	66.2	2577	97.4	16680	23.8	20.1	Mutated
Patient 2	99.3	23152	1.7	118.6	2.95	73.2	Mutated
Patient 3	94.7	22902	43.8	803	24.1	703	Unmutated

Patient 4	82.7	3170	98.7	10760	55.37	3333	Mutated
Patient 5	98.3	6259	89.7	6768	5.6	66.3	Unmutated
Patient 6	98.3	25927	3.15	92	2.07	129	Mutated
Patient 7	99.7	27064	37.7	435	67.1	2002	-
Patient 8	98.3	36711	95.3	7132	90.8	4696	-
Patient 9	99.8	11733	3.4	31.2	0.6	53.6	Mutated
Patient 10	99.8	11693	3.4	32.9	0.6	53.6	Unmutated

B

**miRNA-Seq Circ vs Mig**							
**Sample ID**	**CXCR4 %**	**CXCR4 MFI**	**CD49d %**	**CD49d MFI**	**CD38 %**	**CD38 MFI**	**IGVH Status**
Patient 1	66.2	2577	97.4	16680	23.8	20.1	Mutated
Patient 2	99.3	23152	1.7	118.6	2.95	73.2	Mutated
Patient 3	94.7	22902	43.8	803	24.1	703	Unmutated
Patient 4	82.7	3170	98.7	10760	55.37	3333	Mutated
Patient 5	98.3	6259	89.7	6768	5.6	66.3	Unmutated
Patient 6	98.3	25927	3.15	92	2.07	129	Mutated
Patient 7	96.2	26046	9	0	1.3	164	Mutated
Patient 8	99.7	27064	37.7	435	67.1	2002	-

C

**RNA-Seq Coculture**							
**Sample ID**	**CXCR4 %**	**CXCR4 MFI**	**CD49d %**	**CD49d MFI**	**CD38 %**	**CD38 MFI**	**IGVH Status**
Patient 1	99.6	33640	99.8	8634	52.5	1084	Mutated
Patient 2	98.4	27694	6.3	91.9	21	620	-
Patient 3	82.1	4769	28.7	386	70.9	1754	Mutated
Patient 4	95.2	9845	82.2	3020	42.15	589	Unmutated
Patient 5	82.7	3170	98.7	10760	55.37	3333	Mutated
Patient 6	76.5	2935	0.13	55.8	17.8	557	Mutated
Patient 7	98.4	3316.5	10.2	0	8.7	118.7	-
Patient 8	99.2	22352	2.2	39.1	23.4	498	Mutated

D

**TLR9 Migration**									
**Sample ID**	**CXCR4 %**	**CXCR4 MFI**	**CD49d %**	**CD49d MFI**	**CD38 %**	**CD38 MFI**	**TLR9 %**	**TLR9 MFI**	**IGVH Status**
Patient 1	98.1	5072	61	1193	83.9	2646	91.0	3059	Unmutated
Patient 2	83.2	7727	8.9	305	86.8	5110	74.4	1875	Unmutated
Patient 3	98.4	3317	10.2	0	8.7	119	92.3	3355	-
Patient 4	99.8	11474	1.7	0	0.62	0	85.1	1700	Mutated
Patient 5	99.9	6612	100	19151	98.8	1088	84.3	1796	Mutated
Patient 6	64.1	2291	97.3	16635	19.38	0	89.2	2064	Mutated

E

**Synergy Experiment**							
**Sample ID**	**CXCR4 %**	**CXCR4 MFI**	**CD49d %**	**CD49d MFI**	**CD38 %**	**CD38 MFI**	**IGVH Status**
Patient 1	99	6606	52.7	1580	22.2	359	Unmutated
Patient 2	93.8	3175	35.5	179	54.5	1226	Mutated

Patient 3	89.2	4206	84.8	3917	26.2	296	Unmutated

F

**Invasion Assay**							
**Sample ID**	**CXCR4 %**	**CXCR4 MFI**	**CD49d %**	**CD49d MFI**	**CD38 %**	**CD38 MFI**	**IGVH Status**
Patient 1	99.3	11916	81.7	4232	6.1	934	-
Patient 2	99.9	7657	0	0	47.0	809	Mutated
Patient 3	91.8	4320	84.6	4271	70.4	1944	-
Patient 4	64.1	2290	97.3	16635	19.4	0	Mutated
Patient 5	96.2	26046	9	0	1.3	164	Mutated
Patient 6	76.5	2935	0.13	55.8	17.8	557	Mutated
